# Eosinophilic, polymorphic, and pruritic eruption associated with radiation treated with dupilumab in a patient with Hodgkin lymphoma

**DOI:** 10.1016/j.jdcr.2025.12.045

**Published:** 2026-01-12

**Authors:** Mallory Hartman, Laura Russell, Sagun Goyal, Nicole Burkemper, Gillian Heinecke, Maria Yadira Hurley, Kristin Smith, Emily H. Smith

**Affiliations:** a Saint Louis University School of Medicine, St. Louis, Missouri; b Sisters of St. Mary Healthcare System, Department of Dermatology, Saint Louis University, St. Louis, Missouri; c Sisters of St. Mary Healthcare System, Department of Oncology, Saint Louis University, St. Louis, Missouri

**Keywords:** dupilumab, eosinophilic, polymorphic, pruritic, radiation

## Introduction

Eosinophilic polymorphic and pruritic eruption associated with radiation (EPPER) was first defined in 1999 by Rueda et al^1^ upon observation of patients with cancer experiencing widespread, polymorphic, and pruritic eruptions after undergoing radiation therapy. The histopathologic findings often demonstrate superficial perivascular infiltrates with prominent eosinophils.^1^ Clinically, EPPER can present with erythematous and pruritic papules, vesicles, bullae, nodules, or pustules.^1^, ^2^, ^3^ A majority of the published case reports on this condition have involved female patients, although a few reports have demonstrated EPPER in male patients with prostate cancer and Merkel cell carcinoma.^4^^,^^5^ Rueda et al demonstrated the relative risk of EPPER is radiation dose dependent with the mean radiation dose at onset being 30 Gy. Most reported cases of EPPER involve patients that experienced the eruption while undergoing radiation treatment or within 2 months of completion,^2^, ^3^, ^4^, ^5^, ^6^ although 1 patient experienced EPPER 7 months after completion of radiation.^7^ The eruption typically resolves within 2 to 12 weeks after initiation of treatment with antihistamines, topical or oral steroids, or UV-B phototherapy.^4^ In this case report, we present a 47-year-old man with a history of early relapsing Hodgkin lymphoma who experienced EPPER after 2 sessions of radiation, successfully treated with dupilumab.

## Case report

A 47-year-old man with a history of early relapsing stage IIB Hodgkin lymphoma (nodular sclerosing subtype), status post chemotherapy and autologous stem cell transplant, presented with a widespread pruritic eruption on his arms, legs, buttocks, and lower back after 2 sessions of radiation to his mediastinum for treatment of his relapse. Out of concern for a drug reaction, the patient’s oncologist substituted his *Pneumocystis jirovecii* pneumonia prophylactic therapy of sulfamethoxazole-trimethoprim for atovaquone. He was also prescribed a 6-day course of prednisone, triamcinolone 0.5% ointment, and antihistamines without improvement.

**Fig 1:**
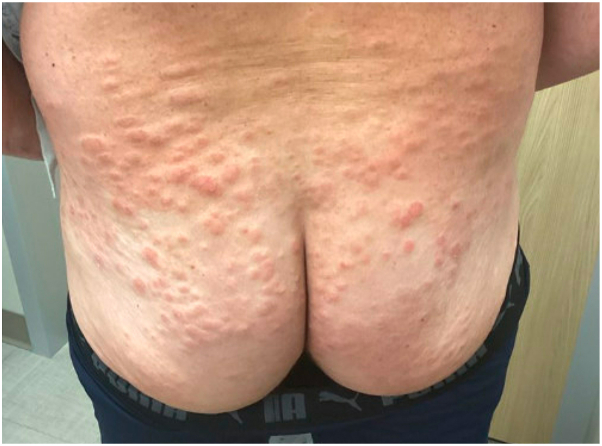
Clinical presentation. Erythematous and flesh-colored edematous and indurated papulonodules scattered across the lower back and buttocks.

**Fig 2:**
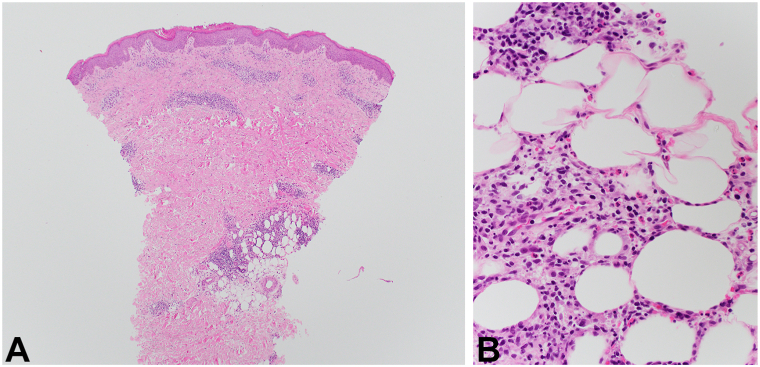
**A,** Histopathologic features. Punch biopsy obtained from the patient’s right thigh demonstrating a superficial and deep perivascular infiltrate. **B,** Histopathologic features. On higher power, a mononuclear infiltrate of lymphocytes and histiocytes, as well as numerous eosinophils is seen among perieccrine and periadnexal adipose tissue extending into the reticular dermis. (Original magnifications: **A,** ×4; **B,** ×40)

On physical examination, erythematous and flesh-colored edematous and indurated papulonodules were observed on the bilateral upper and lower extremities, lower abdomen, lower back, and buttocks (Fig 1). Two 4-mm punch biopsies were obtained. A direct immunofluorescence study was negative for immunoreactant deposition. Histologic examination demonstrated focal parakeratosis, spongiosis, and a superficial and deep perivascular lymphohistiocytic inflammatory infiltrate with eosinophils was seen within the dermis (Fig 2). Review of the patient’s recent and repeated complete blood count tests did not demonstrate peripheral eosinophilia, minimizing concern for hypereosinophilic syndrome. Given the timing of the eruption, a clinicopathologic diagnosis of EPPER was made. The patient was started on triamcinolone 0.1% ointment twice daily to the affected areas, a 4-week prednisone taper, and hydroxyzine 25 mg tablets as needed, which initially resulted in improvement, however, his lesions returned with multiple attempts at a prednisone taper. Given the patient’s need for a steroid-sparing agent and the predominance of eosinophils on his histologic examination, a decision was made—in collaboration with the patient’s oncologist—to begin treatment with dupilumab. He was prescribed the standard dosing regimen of dupilumab with a loading dose of 600 mg followed by 300 mg administered subcutaneously every 2 weeks. The patient subsequently experienced complete resolution of the rash within 2 months of initiating dupilumab and he has not had any flares since.

## Discussion

Unlike acute and chronic radiation dermatitis, which typically affects radiated skin exclusively, EPPER often involves both irradiated and nonirradiated skin. Given the clinical presentation of EPPER can be somewhat variable, and affected patients are often immunocompromised, the differential diagnosis can be quite broad. Clinically, the differential diagnosis for this patient’s eruption included histiocytoid Sweet syndrome, granulomatous drug reaction, and secondary syphilis. These conditions were largely excluded by histopathologic evaluation and the distinct clinical timing of the eruption after 2 sessions of radiation therapy. Eosinophilic dermatosis of hematologic malignancy was considered; however, the temporal association of the development of his eruption shortly after starting radiation therapy, favored EPPER. Additionally, the patient had no prior similar eruptions when he had documented active disease and a positron emission tomography scan confirmed remission shortly after his initial diagnosis. Parasitic infection was another pertinent diagnostic consideration in this immunocompromised patient presenting with an eosinophilic eruption, however, this diagnosis was not favored in the absence of gastrointestinal symptoms and peripheral eosinophilia on routine laboratory monitoring. Other infectious etiologies were ruled out via histologic examination as *Treponema pallidum* immunostaining, Grocott’s methenamine silver stain, Gram stain, and Fite stain were all negative.

Various reports have demonstrated that EPPER typically self-resolves or responds favorably to topical or oral steroids, UV-B treatments, and antihistamines.^1^^,^^3^, ^4^, ^5^, ^6^, ^7^ This case is unusual because the patient’s eruption was persistent on his legs despite use of these treatments, however, he achieved complete resolution with use of dupilumab. A rare association between dupilumab and the unmasking or progression of lymphoproliferative disorders, specifically cutaneous T-cell lymphoma has been observed; however, this pattern has occurred largely in patients who were initially misdiagnosed with atopic dermatitis or who had a known history of cutaneous T-cell lymphoma.^8^ Such cases typically manifest as lack of clinical improvement, disease progression, or new morphologic changes while on dupilumab.^8^ To our knowledge, there are no reports of unmasking of systemic lymphoproliferative disorders in patients on dupilumab, as would be the greater concern for this patient. He will continue dupilumab indefinitely, with ongoing collaboration with his oncologist and regular follow-ups to monitor for signs of recurrence.

Although the pathophysiology of EPPER remains poorly understood, this patient’s positive response to dupilumab reaffirms the important role of eosinophils within this condition and perhaps more specifically, the role of cytokines interleukin 4 and interleukin 13 and the type 2 inflammation pathway.^9^ It is plausible that the patient’s autologous stem cell transplant may have also played a role in the development of his persistent EPPER as a previous study has shown that autologous stem cell transplants in patients with non-Hodgkin lymphoma can alter the balance between T helper 1 and T helper 2 cells.^10^ We recommend dermatologists work in conjunction with oncologists to consider dupilumab as a treatment option in patients with persistent EPPER or in patients who require a steroid-sparing agent. EPPER should also be considered in the differential diagnosis of a patient presenting with a diffuse eruption early in the course of radiation treatment.

## Conflicts of interest

None disclosed.
